# Development and validation of a preoperative CT-based radiomic nomogram to predict pathology invasiveness in patients with a solitary pulmonary nodule: a machine learning approach, multicenter, diagnostic study

**DOI:** 10.1007/s00330-021-08268-z

**Published:** 2021-10-16

**Authors:** Luyu Huang, Weihuan Lin, Daipeng Xie, Yunfang Yu, Hanbo Cao, Guoqing Liao, Shaowei Wu, Lintong Yao, Zhaoyu Wang, Mei Wang, Siyun Wang, Guangyi Wang, Dongkun Zhang, Su Yao, Zifan He, William Chi-Shing Cho, Duo Chen, Zhengjie Zhang, Wanshan Li, Guibin Qiao, Lawrence Wing-Chi Chan, Haiyu Zhou

**Affiliations:** 1 Division of Thoracic Surgery, Guangdong Provincial People’s Hospital & Guangdong Academy of Medical Sciences, The Second School of Clinical Medicine, Southern Medical University, Shantou University Medical College, Guangzhou, China; 2 Guangdong Provincial Key Laboratory of Malignant Tumor Epigenetics and Gene Regulation, Department of Medical Oncology, Phase I Clinical Trial Centre, Sun Yat-Sen Memorial Hospital, Sun Yat-Sen University, Guangzhou, China; 3 AI & Digital Media Concentration Program, Division of Science and Technology, Beijing Normal University-Hong Kong Baptist University United International College, Zhuhai, China; 4 Department of Radiology, Zhoushan Hospital, Zhoushan City, Zhejiang Province China; 5 Department of Pathology, Zhoushan Hospital, Zhoushan City, Zhejiang Province China; 6 Department of Radiology, Department of PET Center, Guangdong Provincial People’s Hospital & Guangdong Academy of Medical Sciences, Guangzhou, China; 7 Department of Pathology, Guangdong Provincial People’s Hospital & Guangdong Academy of Medical Sciences, Guangzhou, China; 8 Department of Clinical Oncology, Queen Elizabeth Hospital, Hong Kong, China; 9 Department of Respiratory and Critical Care Medicine, Beijing Institute of Respiratory Medicine, Beijing Chao-Yang Hospital, Capital Medical University, Beijing, China; 10 Clinical Medicine, Zhongshan School of Medicine, Yat-Sen University, Guangzhou, China; 11 Department of Health Technology and Informatics, The Hong Kong Polytechnic University, Hong Kong, China

**Keywords:** Solitary pulmonary nodule, Nomograms, Lung, Algorithms, Tomography, X-ray computed

## Abstract

**Objectives:**

To develop and validate a preoperative CT-based nomogram combined with radiomic and clinical–radiological signatures to distinguish preinvasive lesions from pulmonary invasive lesions.

**Methods:**

This was a retrospective, diagnostic study conducted from August 1, 2018, to May 1, 2020, at three centers. Patients with a solitary pulmonary nodule were enrolled in the GDPH center and were divided into two groups (7:3) randomly: development (*n* = 149) and internal validation (*n* = 54). The SYSMH center and the ZSLC Center formed an external validation cohort of 170 patients. The least absolute shrinkage and selection operator (LASSO) algorithm and logistic regression analysis were used to feature signatures and transform them into models.

**Results:**

The study comprised 373 individuals from three independent centers (female: 225/373, 60.3%; median [IQR] age, 57.0 [48.0–65.0] years). The AUCs for the combined radiomic signature selected from the nodular area and the perinodular area were 0.93, 0.91, and 0.90 in the three cohorts. The nomogram combining the clinical and combined radiomic signatures could accurately predict interstitial invasion in patients with a solitary pulmonary nodule (AUC, 0.94, 0.90, 0.92) in the three cohorts, respectively. The radiomic nomogram outperformed any clinical or radiomic signature in terms of clinical predictive abilities, according to a decision curve analysis and the Akaike information criteria.

**Conclusions:**

This study demonstrated that a nomogram constructed by identified clinical–radiological signatures and combined radiomic signatures has the potential to precisely predict pathology invasiveness.

**Key Points:**

*• The radiomic signature from the perinodular area has the potential to predict pathology invasiveness of the solitary pulmonary nodule.*

*• The new radiomic nomogram was useful in clinical decision-making associated with personalized surgical intervention and therapeutic regimen selection in patients with early-stage non-small-cell lung cancer.*

**Supplementary Information:**

The online version contains supplementary material available at 10.1007/s00330-021-08268-z.

## Introduction

Low-dose computed tomography (LDCT) screening has been shown to reduce lung cancer mortality in a high-risk group [1]. In lung cancer screening trials, the nodule prevalence (%) is 33% on average, while only 1.4% of the detected nodules are diagnosed as lung cancer. How to select patients with malignant pulmonary nodules for timely intervention has become a major challenge.

Numerous appropriate follow-up protocols are utilized to manage these pulmonary nodules detected by CT screening. For indeterminate nodules, the Fleischner Society guidelines [2] and the Lung CT Screening Reporting and Data System (Lung-RADS) prescribe a CT screening after a particular time interval based on nodule size. However, recommendations from the British Thoracic Society (BTS) guidelines [3] reduce the need for follow-up imaging for patients with nodules of < 5 mm diameter or < 80 mm^3^, and a reduction of the follow-up period to 1 year for solid pulmonary nodules (SPN). However, the awareness of recommendations and the management choices in clinical practice have exhibited heterogeneity between radiologists and pulmonologists [4].

Differentiating pathology types between a pulmonary precancerous lesion (i.e., atypical adenomatous hyperplasia and adenocarcinoma in situ (AAH/AIS)) and early-stage invasive adenocarcinoma (IAC) leads to vastly divergent prognoses after standard thoracic surgery [5–7]. Minimally invasive adenocarcinoma (MIA) is a small, solitary adenocarcinoma with mostly lepidic growth and an invasion smaller than 5 mm at its largest dimension at any one point, whereas invasive adenocarcinoma involves growths larger than 5 mm [5]. It is challenging to identify the pathological nature of suspected malignant pulmonary nodules through visual assessment with CT scans because of the considerable overlap in morphologic features between them, such as pleural tags, spiculation, and lobulation [8].

Models including machine learning and artificial neural networks have been applied in lung cancer diagnosis [9–12], and excellent identification efficiency and accuracy have been achieved according to internal data. However, these tools suffer from limited external validity, overfitting, and unexplainable results [13]. Radiomics provides a noninvasive approach and is more promising in its sensitivity, selectivity, and experimental feasibility for disease diagnosis, tumor staging, and patient prognosis [14–17].

Tumor-infiltrating lymphocytes and tumor-associated macrophages were observed to be distributed at the edge of the invasion lesions (ILs) in the pathological map [18] and be associated with the likelihood of metastasis [19]. The perinodular parenchyma may be considered to represent the tumor microenvironment and has biological importance in defining tumor behavior, including cell migration, stromal inflammation, immune infiltration, and vascularization [20, 21]. We assumed that radiomic signatures from perinodular areas might provide a preoperative reference for the accurate prediction of pathological invasiveness in solitary pulmonary nodules and for guiding surgical methods and the extent of resection.

Herein, we developed and validated a nomogram based on clinical–radiological and radiomic signatures from nodular and perinodular areas for preoperative prediction of pathological invasiveness in patients with a solitary pulmonary nodule using data from a multicenter study.

## Methods

### Study design and patients

In this multicenter, retrospective, diagnostic study, patients with a solitary pulmonary nodule were recruited from three independent centers (Guangdong Provincial People’s Hospital, Guangdong Province, China, named as the GDPH center; Sun Yat-sen Memorial Hospital of Sun Yat-sen University, Guangdong Province, China, named as the SYSMH center; Zhoushan Lung Cancer Institution, Zhejiang Province, China, named as the ZSLC center) during the period of August 1, 2018, to May 1, 2020. Information about the three institutions that participated in this study is shown in eTable 1.

**Fig. 1:**
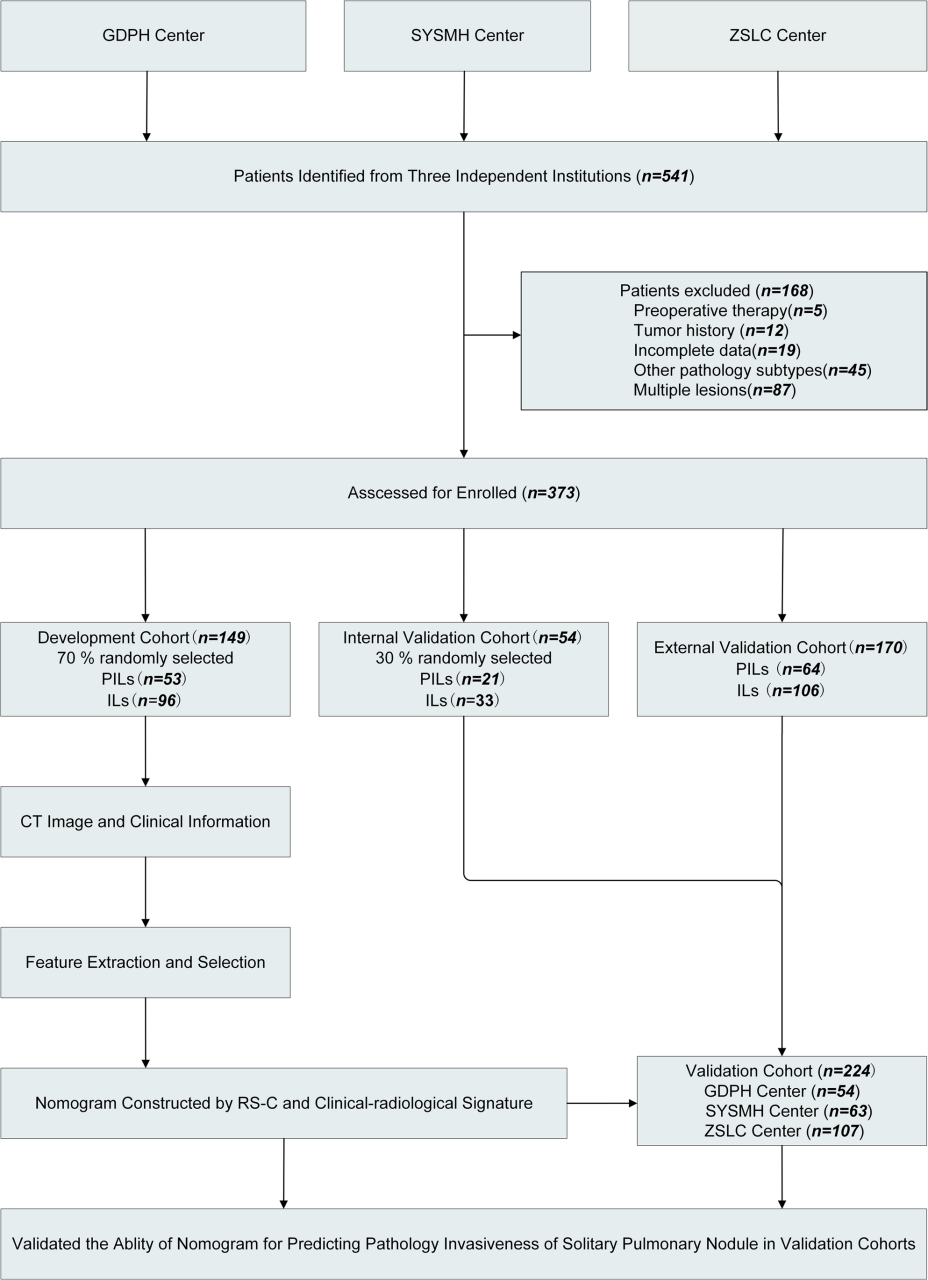
Patient recruitment process at three centers. GDPH center, Guangdong Provincial people’ s Hospital; SYSMH center, Sun Yat-Sen Memorial Hospital of Sun Yat-Sen University; ZSLC center, Zhoushan Lung Cancer Institution; CT, computed tomography; PILs, pre-invasive lesions; ILs, invasion lesions; RS-C, combined radiomic signature selected from the nodular area and perinodular area

The inclusion and exclusion criteria were applied at the three centers, and 373 patients from the 571 recruited patients were finally enrolled after the application of the exclusion criteria. The patients (*N* = 203) enrolled at the GDPH center from March 1, 2015, to December 31, 2019, were divided into two cohorts: The development cohort comprised 149 patients (73.4%) randomly selected by a computer algorithm in a ratio of 7:3, and the validation cohort comprised 54 patients (26.6%). The SYSMH center (*N* = 63) and the ZSLC center (*N* = 107) cooperatively formed an external validation cohort of 170 patients from December 18, 2012, to July 30, 2019, and January 1, 2019, to December 30, 2019. Figure 1 presents the exclusion criteria and the patient recruitment process.

The following were the criteria for inclusion: (I) patients ≥ 18 years of age who underwent CT screening and were diagnosed with SPN for the first time, (II) patients who underwent preoperative enhanced chest CT scans (within 3 months), (III) pathologically confirmed precancerous lesions (AAH/AIS) or early-stage lung adenocarcinoma (MIA/IAC), and (IV) lesions smaller than 30 mm without distant metastases, or lymph node involvement.

The exclusion criteria were (I) preoperative therapy (neoadjuvant chemotherapy or radiotherapy), (II) a history of previous lung tumor diseases or (III) past/present history of other malignant tumors, and (IV) incomplete clinical information or unavailable standard enhanced chest imaging data.

Because of the retrospective nature of this study, the institutional review board waived informed patient consent. The study protocol was approved by academic ethics committees and conducted according to the Declaration of Helsinki and Good Clinical Practice guidelines and was registered with ClinicalTrials.gov (registration number NCT04452058).

### Image review and feature extraction

The Picture Archiving and Communication System (PACS) was used to retrieve preoperative CT images from three centers, and all researchers assessed the initial screening of image data. The CT protocol is described in detail in eTable 2.

The regions of interest (ROIs) in the pulmonary nodular area (ROI-1) were all manually refined by one researcher (W.H.L.) slice by slice in three orthogonal planes (axial, coronal, and sagittal) under the guidance of two senior radiologists with 13 years (S.Y.W.) and 17 years (G.Y.W.) of experience in chest CT interpretation. Other irrelevant components, such as air, peripheral vessels, normal tissue, ribs, pleura, and surrounding organs, were removed by the researchers to avoid interference. The 3D Slicer program was used to semi-automatically segment the perinodular area (ROI-2, including the perinodular parenchymal representing a 5-mm extension outward) (https://www.slicer.org/, version 4.10.2) [22]. The disagreement was resolved by discussion among senior researchers, including two radiologists and three thoracic surgeons (Q.G.B., Z.H.Y., and Z.D.K.).

All assessors were blind to the final pathology diagnostics which were reviewed by a senior pathologist (S.Y.) using the 2017 8th TNM staging system and the 2011 International Association for the Study of Lung Cancer/American Thoracic Society/European Respiratory Society (IASLC/ATS/ERS) classification for pathological staging and pathological grading after thoracic surgery, respectively [5, 23].

After the ROI-1 and ROI-2 were segmented and reconstructed, the volume of interest (VOI-1 and VOI-2) images (DICOM format) were transferred to the SlicerRadiomics code using an in-house texture extraction platform based on the Python package PyRadiomics.

**Fig. 2:**
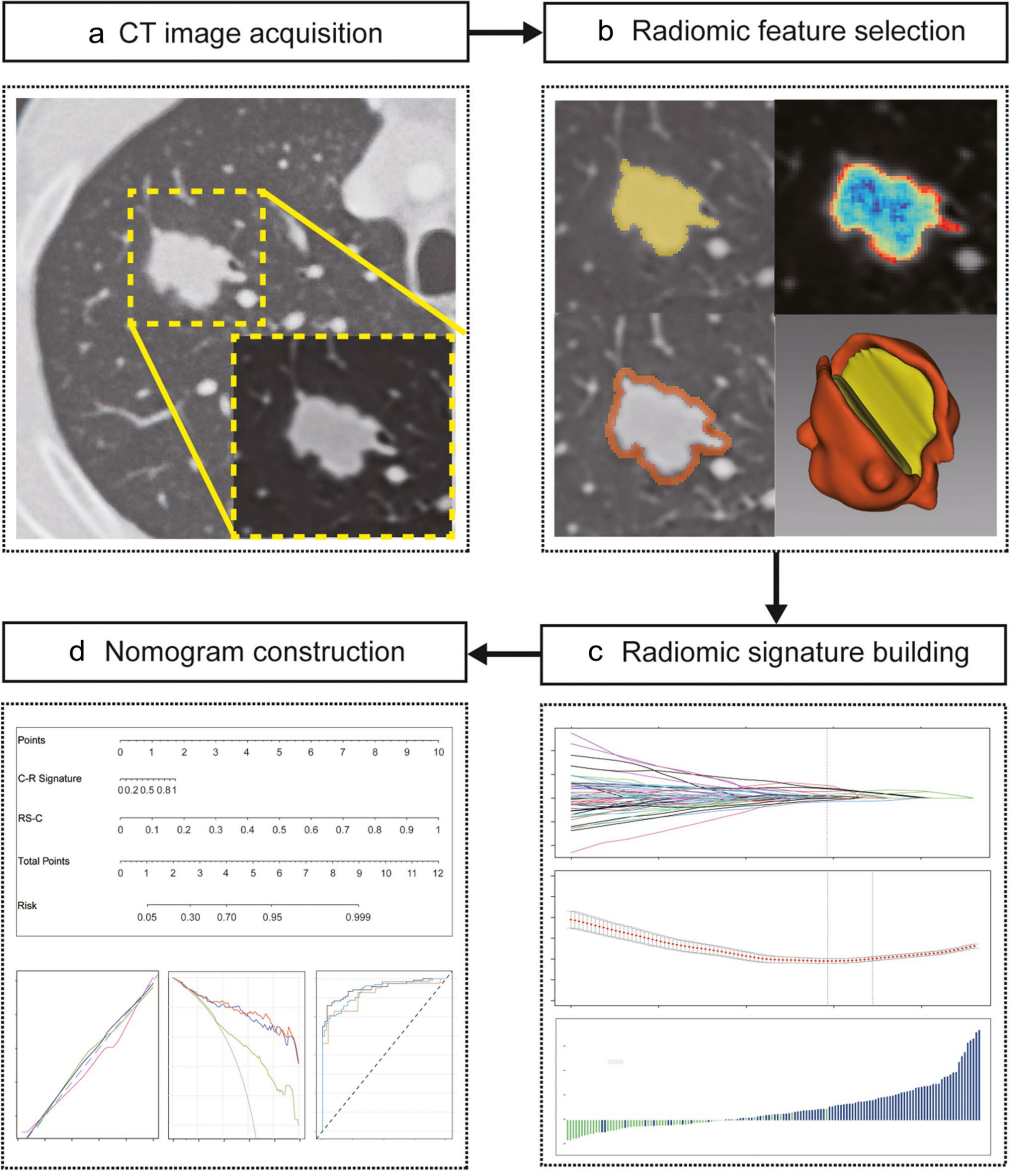
Overall radiomic workflow and pipeline in this study. **a** CT image (transverse section) in a 58-year-old male patient with a 1.5-cm solitary pulmonary nodule in the right upper lung (dotted box) on contrast-enhanced CT and biopsy confirmed as lung adenocarcinoma. **b** Two regions of interest (ROIs) were constructed into volumes of interests (VOIs), and radiomic features were extracted from two VOIs. **c** Radiomic features were selected by the LASSO algorithm and constructed into a radiomic signature. **d** Discrimination and calibration of the nomogram which was formed by the clinical–radiological and combined radiomic signatures

There are 1722 quantitative radiomic features in all, which include first-order statistics, shape, gray-level co-occurrence matrix (GLCM), gray-level size zone matrix (GLSZM), gray-level dependence matrix (GLDM), and neighborhood gray-tone difference matrix (NGTDM), which were extracted from two segmented regions (VOI-1 and VOI-2). These features were used for further analysis and regression modeling. The same image segmentation process and feature extraction were conducted among 30 SPNs in the cohorts after 3 months. More information about the standard radiomic workflow and model construction is shown in Fig. 2.

### Development of the radiomic signatures

High-dimensional imaging data is featured from the two VOIs by the LASSO algorithm (eFigure 1 in the Supplementary materials). By linear combination, the most useful predicted combination of data was used to create two radiomic signatures (RS1 for VOI-1 and RS2 for VOI-2).

The final radiomic signature was combined with two radiomic signatures obtained by logistics regression. The tenfold cross-validation was implemented to avoid overfitting. Based on the combined radiomic signature (RS-C), the radiomic score was calculated and presented in the development and two validation cohorts.

### Development of the clinical–radiological signature and nomogram

Baseline clinical data were obtained from medical records. The researchers also recorded several radiological feature descriptors of each pulmonary nodule, such as the size, number, location, border, and internal characteristics (e.g., density and consolidation tumor ratio (CTR)), and any disagreement was resolved through consultation. The densities of pulmonary nodules were described using terminology derived from the BTS guidelines [3].

After the analysis, significant risk factors were used to build a clinical–radiological signature. This signature was combined with the final radiomic signature (RS-C) to form a nomogram using logistic regression.

### Statistical analysis

Normalization was performed on radiomic features using a *z*-score transformation. To investigate differences in categorical variables, the chi-square test was used. The differences in continuous variables between PILs and early-stage pulmonary interstitium ILs were compared using a two-sample *t* test.

Univariate logistic regression analysis was used to select the independent clinical and radiological prognostic factors in the internal cohort. Significant risk factors were then introduced into stepwise logistical regression analyses to build a clinical–radiological signature. To visualize the results of the multivariable logistic regression analysis for risk stratification of pathological invasiveness, a nomogram based on both the clinical–radiological signature and the combination radiomic signature was created. Intrarater agreement in radiomic features between two times ROI segmentation was assessed using the two-way random ICC model.

To evaluate the performance of models, a receiver operating characteristic (ROC) analysis was done, and the accuracy, sensitivity, specificity, negative predictive value (NPV), and positive predictive value (PPV) were calculated. The DeLong test compared the nomogram to other models in the development cohort in terms of the area under the ROC curve (AUC).

The Akaike information criterion (AIC) [24] was used to compare and rank multiple competing models and emphasize the comparison of the goodness of fit of the competing models while considering the principle of parsimony. We chose the model with the lowest AIC value (representing the “best-approximating model”) in this study.

The utility and clinical value of models can be evaluated using decision curve analysis (DCA) [25], which determines the net benefit for patients at each threshold probability. The calibration of the nomogram was assessed using the Hosmer–Lemeshow test and calibration curves in the three cohorts. Two-sided *p* values < 0.05 indicated statistical significance. The packages of GLMNET were run, and statistical analysis was performed using R software (version 3.6.2; http://www.Rproject.org).

## Results

### Participants

The imaging of 373 preoperative patients with a solitary pulmonary nodule was collected from three independent institutions in China. The development cohort included 149 patients from the GDPH center (female, 61.7%; median [IQR] age, 59.0 [49.0 to 66.0] years), and the internal validation cohorts included 54 patients (female, 57.4%; median [IQR] age, 54.7 [46.0 to 63.8] years). The external validation cohort from the SYSMH and ZSLC centers included 170 patients (female, 60.0%; median [IQR] age, 57 [48.3 to 65.0] years).

In the PIL group, 35.6% (53/149) of the patients were diagnosed with PILs (AAH/AIS) in the development cohort, 38.9% (21/54) were diagnosed with PILs in the internal validation cohort, and 37.6% (64/170) were diagnosed with PILs in the external validation cohort. Table 1 shows the baseline characteristics of the patients in the development and two validation cohorts.

**Table 1:** Characteristic baseline of patients in cohorts

Variable	Development cohort (*N* = 149)	Internal validation cohort (*N* = 54)	External validation cohort (*N* = 170)	Total cohort (*N* = 373)
Group, no. (%)				
Pre-invasive lesions	53 (35.6)	21 (38.9)	64 (37.6)	138 (37.0)
Invasive lesions	96 (64.4)	33 (61.1)	106 (62.4)	235 (63.0)
Age at diagnosis, years, no. (%)				
Mean (SD)	57.5 (12.7)	54.7 (12.0)	56.9 (12.0)	56.8 (12.3)
Median (IQR)	59.0 [49.0, 66.0]	55.0 [46.0, 63.8]	57.0 [48.3, 65.0]	57.0 [48.0, 65.0]
Range	[29.0, 86.0]	[27.0, 80.0]	[21.0, 80.0]	[21.0, 86.0]
< 60 y	77 (51.7)	36 (66.7)	102 (60.0)	215 (57.6)
≥ 60 y	72 (48.3)	18 (33.3)	68 (40.0)	158 (42.4)
Gender, no. (%)				
Female	92 (61.7)	31 (57.4)	102 (60.0)	225 (60.3)
Male	57 (38.3)	23 (42.6)	68 (40.0)	148 (39.7)
Primary site of tumor, no. (%)				
LLL	23 (15.4)	9 (16.7)	29 (17.1)	61 (16.4)
LUL	35 (23.5)	13 (24.1)	39 (22.9)	87 (23.3)
RLL	27 (18.1)	12 (22.2)	37 (21.8)	76 (20.4)
RML	11 (7.4)	3 (5.6)	11 (6.5)	25 (6.7)
RUL	53 (35.6)	17 (31.5)	54 (31.8)	124 (33.2)
Density, no. (%)				
pGGN	58 (38.9)	26 (48.1)	47 (27.6)	131 (35.1)
PSN	69 (46.3)	23 (42.6)	64 (37.6)	156 (41.8)
Solid	22 (14.8)	5 (9.3)	59 (34.7)	86 (23.1)
Pleural retraction, no. (%)				
No	110 (73.8)	38 (70.4)	111 (65.3)	259 (69.4)
Yes	39 (26.2)	16 (29.6)	59 (34.7)	114 (30.6)
Bubble sign, no. (%)				
No	130 (87.2)	46 (85.2)	144 (84.7)	320 (85.8)
Yes	19 (12.8)	8 (14.8)	26(15.3)	53 (14.2)
Shape, no. (%)				
Round or oval	84 (56.4)	26 (48.1)	80 (47.1)	190 (50.9)
Irregular	65 (43.6)	28 (51.9)	90 (52.9)	183 (49.1)
Clear margin, no. (%)				
No	118 (79.2)	44 (81.5)	103 (60.6)	265 (71.0)
Yes	31 (20.8)	10 (18.5)	67 (39.4)	108 (29.0)
Lobulated border, no. (%)				
No	97 (65.1)	30 (55.6)	78 (45.9)	205 (55.0)
Yes	52 (34.9)	24 (44.4)	92 (54.1)	168 (45.0)

Abbreviations: *SD* standard deviation, *IQR* interquartile range, *PILs* pre-invasive lesions, *ILs* invasive lesions, *LLL* left lower lobe, *LUL* left upper lobe, *RLL* right lower lobe, *RML* right middle lobe, *RUL* right upper lobe, *pGGN* pure ground-glass nodule, *PSN* part-solid nodule

### Validation of the radiomic signatures

In total, 1722 radiomic features were extracted from two VOIs (RS1: four features for VOI-1, RS2: eight features for VOI-2) and were selected by the LASSO algorithm. Moreover, RS1and RS2 were combined into a final radiomic signature (RS-C) using logistic regression, and the radiomic score calculation formula was presented in eTable 3 in Supplementary materials.

The radiomic score for each patient was significantly different between the PIL group (AAH/AIS) and IL group (MIA/IAC) in three cohorts (*p* < 0.001 for eFigure 2A; *p* = 0.003 for eFigure 2B; *p* < 0.001 for eFigure 2C, eTable 4 in the Supplementary materials). The mean value of the radiomic score for patients in the IL group (MIA/IAC) was significantly higher in both the development and two validation cohorts (7.28, 7.45, and 8.34, respectively) compared with the patients in the PIL group (AAH/AIS) (− 1.24, − 1.06, and − 2.59, respectively).

The AUC for the RS1 was 0.83 (95% CI, 0.76 to 0.89) in the development cohort, 0.85 (95% CI, 0.71 to 0.93) in the internal validation cohort, and 0.88 (95% CI, 0.83 to 0.93) in the external validation cohort (eFigure 3A in the Supplementary materials). In the development cohort, the AUC for the RS2 was 0.92 (95% CI, 0.86 to 0.95), and in the internal and external validation cohorts, it was 0.89 (95% CI, 0.80 to 0.98), 0.89 (95% CI, 0.84 to 0.94). (eFigure 3B in the Supplementary materials).

Among all radiomic-related signatures in the development and two validation cohorts, the RS-C had the greatest AUCs of 0.93 (95% CI, 0.89 to 0.97), 0.91 (95% CI, 0.83 to 0.98), and 0.90 (95% CI, 0.85 to 0.94) (eFigure 3C in the Supplementary materials). The two-way random ICC model was applied to measure the reliability of the radiomic features between two-times image segmentation and feature extraction process. The agreement levels are defined regarding ICC values: excellent (ICC ≥ 0.81), good (0.61 < ICC < 0.8), moderate (0.41 < ICC < 0.60), and poor (ICC ≤ 0.40). eTable 5 summarizes the results of the intrarater agreement analysis. Radiomic features in the RS1 show excellent intrarater reliability (ICC = 0.92 to 0.99) between this process, and the high ICC values for radiomic feature in the RS2 ranging from good (ICC = 0.74; 95% CI, 0.521 to 0.872) to excellent (ICC = 0.98; 95% CI, 0.949 to 0.989).

### Validation of the clinical–radiological signature

Clinical–radiological characteristics, including density (part-solid nodule (PSN)/solid nodule), pleural retraction, irregular shape, lobulated borders, CTR ≥ 0.5, and blurred margins, were significantly associated with pathology invasiveness after the univariate analysis (*p* < 0.05; eTable 6 in the Supplementary materials), and four of these characteristics (PSN/solid nodule, irregular shape, pleural retraction, and blurred margins) were selected using the stepwise logistic regression model to form the clinical–radiological signature (eTable 3 in the Supplementary materials).

Based on the ROC analysis, the AUCs for the clinical–radiological signature were 0.79 (95% CI, 0.72 to 0.89), 0.79 (95% CI, 0.67 to 0.92), and 0.88 (95% CI, 0.83 to 0.93) (eFigure 3D in the Supplementary materials) in the development, internal, and external validation cohorts, respectively.

### Validation, calibration, and discrimination of the nomogram

**Fig. 3:**
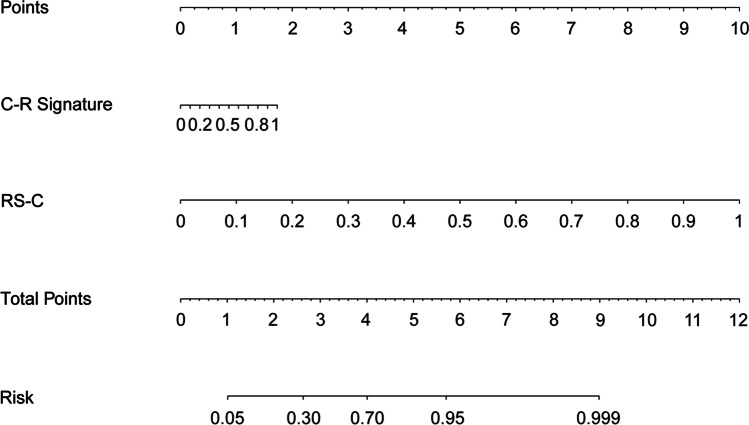
Nomogram based on the radiomic and clinical–radiological signatures. The nomogram based on RS-C and the clinical–radiological signature to predict pathology invasiveness. RS-C, combined radiomic signature selected from the nodular area and perinodular area; C-R, clinical–radiological

To develop a clinically applicable approach that could predict pathological invasiveness in patients with a solitary pulmonary nodule, we constructed a radiomic nomogram that considers the clinical–radiological and radiomic signatures (Fig. 3). The multivariate logistic regression analysis showed that the clinical–radiological signature (odds ratio (OR) = 1.60; 95% CI, 1.03 to 2.59; *p* = 0.04) and the radiomic signature (odds ratio (OR) = 2.43; 95% CI, 1.76 to 3.66; *p* < 0.001) represented independent predictors in the nomogram (eTable 7 in the Supplementary materials).

As shown in the nomogram (Fig. 3), when compared to the clinical–radiological signature, the radiomic signature accounted for the most significant proportion, making it the cardinal biomarker for distinguishing PILs from early-stage ILs. Based on the obtained features, the clinical–radiological signature and combined radiomic signature could be calculated using the formula. The value assigned to each signature was scored on a point scale from 0 to 10. By adding the scores for each signature, one can obtain a total score. The risk of this solitary pulmonary nodule having pulmonary interstitial invasion can be predicted by projecting the score to the bottom risk axis.

**Fig. 4:**
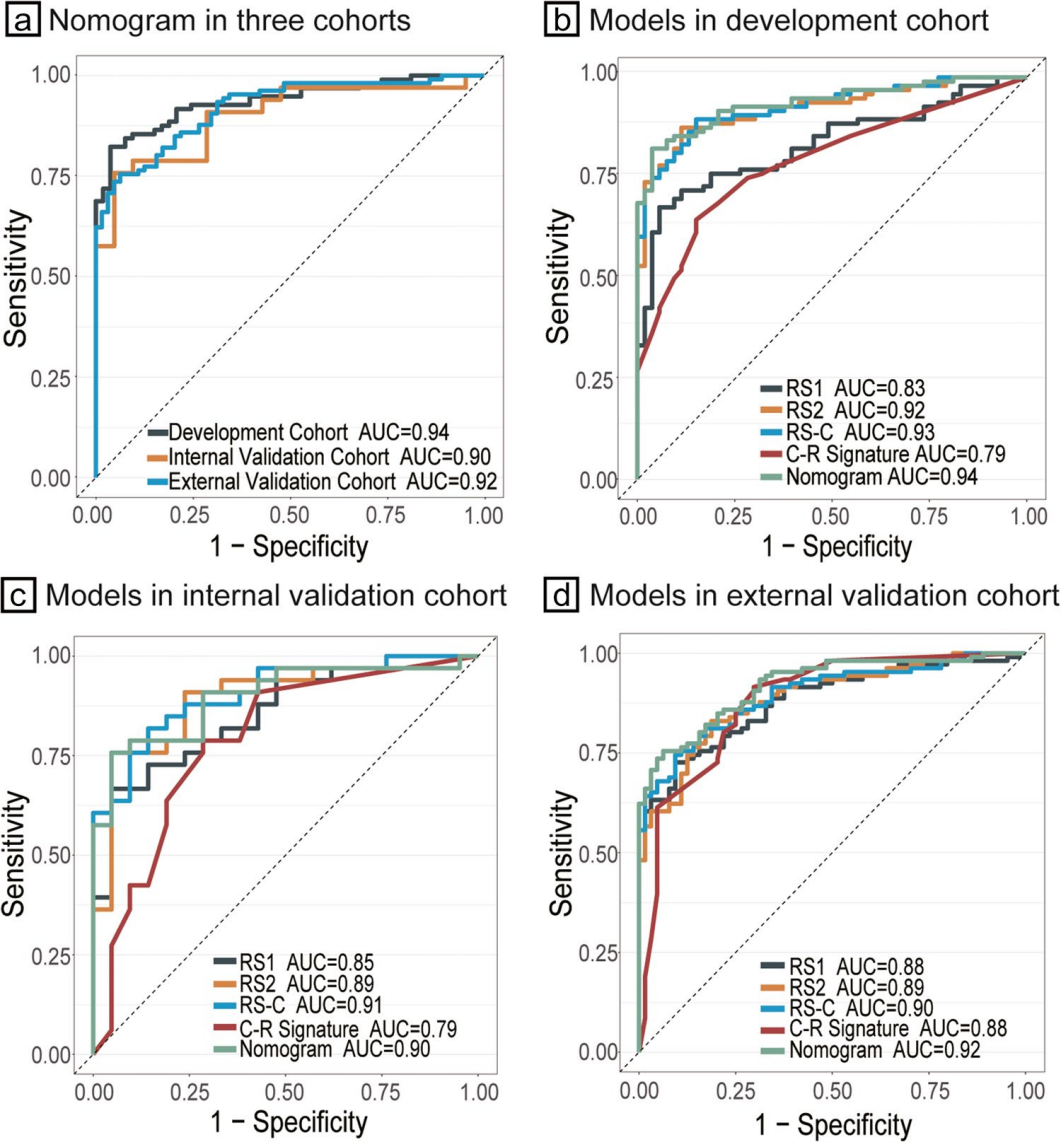
ROC curves of the nomogram and models in the development and validation cohorts. **a** ROC curves of the nomogram in the development and validation cohorts; **b** ROC curves of five models in the development cohort; **c** ROC curves of five models in the internal validation cohorts; **d** ROC curves of five models in the external validation cohorts. ROC, receiver operating characteristic; RS1, radiomic signature selected from the nodular area; RS2, radiomic signature selected from the perinodular area; RS-C, combined radiomic signatures selected from the nodular area and perinodular area; C-R, clinical–radiological

The nomogram formed by the clinical–radiological and combined radiomic signatures performed better than any isolated signatures. The nomogram achieved an excellent predictive value, with an AUC test of 0.94 (95% CI, 0.90 to 0.97) in the development cohort, which achieved better discriminatory performance than the radiomic signatures and clinical–radiological signature (Fig. 4b). Similar findings of model comparisons were also observed in the two validation cohorts (Fig. 4c, d). In the two validation cohorts, the nomogram also yielded high AUCs of 0.90 (95% CI, 0.81 to 0.98) and 0.92 (95% CI, 0.88 to 0.92) (Fig. 4a). Moreover, the accuracy, specificity, and PPV of the nomogram were higher than 80.0% in the three cohorts (Table 2).

**Table 2:** Performance evaluation of the models in the development and two validation cohorts

Cohort	Signature	Signature performance					
		Sensitivity	Specificity	Accuracy	PPV	NPV	AUC (95% CI)
Development cohort	RS1	0.68	0.94	0.77	0.96	0.62	0.83 (0.76–0.89)
	RS2	0.88	0.89	0.88	0.88	0.80	0.92 (0.86–0.95)
	RS-C	0.90	0.85	0.88	0.92	0.82	0.93 (0.89–0.97)
	C-R signature	0.65	0.85	0.72	0.89	0.57	0.79 (0.72–0.86)
	Nomogram	0.82	0.96	0.87	0.98	0.75	0.94 (0.90–0.97)
Internal validation cohort	RS1	0.67	0.95	0.78	0.96	0.65	0.85 (0.71–0.93)
	RS2	0.91	0.76	0.85	0.86	0.84	0.89 (0.80–0.98)
	RS-C	0.82	0.86	0.83	0.90	0.75	0.91 (0.83–0.98)
	C-R signature	0.79	0.71	0.76	0.81	0.68	0.79 (0.66–0.92)
	Nomogram	0.76	0.95	0.83	0.96	0.71	0.90 (0.81–0.98)
External validation cohort	RS1	0.73	0.91	0.79	0.93	0.67	0.88 (0.83–0.93)
	RS2	0.83	0.81	0.82	0.88	0.74	0.89 (0.84–0.94)
	RS-C	0.75	0.91	0.81	0.93	0.68	0.90 (0.85–0.94)
	C-R signature	0.92	0.70	0.84	0.84	0.83	0.88 (0.83–0.93)
	Nomogram	0.76	0.94	0.82	0.95	0.70	0.92 (0.88–0.96)

Abbreviations: *PPV* positive predictive values, *NPV* negative predictive values, *AUC* area under the receiver operating characteristics curve, *CI* confidence interval, *RS1* radiomic signature selected from the nodular area, *RS2* radiomic signature selected from the perinodular area, *RS-C* combined radiomic signature selected from the nodular area and perinodular area, *C-R* clinical–radiological

In order to prove that the nomogram model also has a good discriminatory performance among the SPNs with different densities (pure ground-glass nodule (pGGN), PSN, solid), we conducted a subgroup analysis in this research. In the subgroup of pGGN, the nomogram showed high AUCs of 0.87 (95% CI, 0.79 to 0.95), 0.92 (95% CI, 0.84 to 0.99), and 0.89 (95% CI, 0.83 to 0.95) (eFigure 4 in the Supplementary materials) in the internal, external, and total cohorts, respectively. eTable 8 shows the performance evaluation of the nomogram in subgroup analysis. Subgroup analyses also detected high discrimination of the nomogram in the PSN subgroups (AUC = 0.93, 0.81, and 0.89) and the solid group (AUC = 0.96, 0.93, and 0.94) in cohorts.

The DeLong test was performed on the ROC curves of five models among the AUCs in the development cohort. The differences were statistically significant between the nomogram and RS1 and the nomogram and the clinical–radiological signature, with *p* = 0.005 and *p* < 0.001, respectively (eTable 9 in the Supplementary materials). The clinical–radiological signature achieved the highest AIC value at 159.36 among all prediction models in eTable 9. The AIC of the nomogram (121.68) was similar to that of the radiomic signature (121.0) and RS2 (117.62), which was less than the AIC of RS1 (149.83). Based on the overall consideration of the AIC and ROC curves, the nomogram model proved to have excellent goodness of fit and parsimony.

Moreover, the nomogram calibration curve in cohorts indicated a good agreement between the nomogram prediction and actual observation. The Hosmer–Lemeshow test revealed that the nomogram was well fitting, with a nonsignificant difference (*p* > 0.05) (eFigure 5 in the Supplementary materials).

**Fig. 5:**
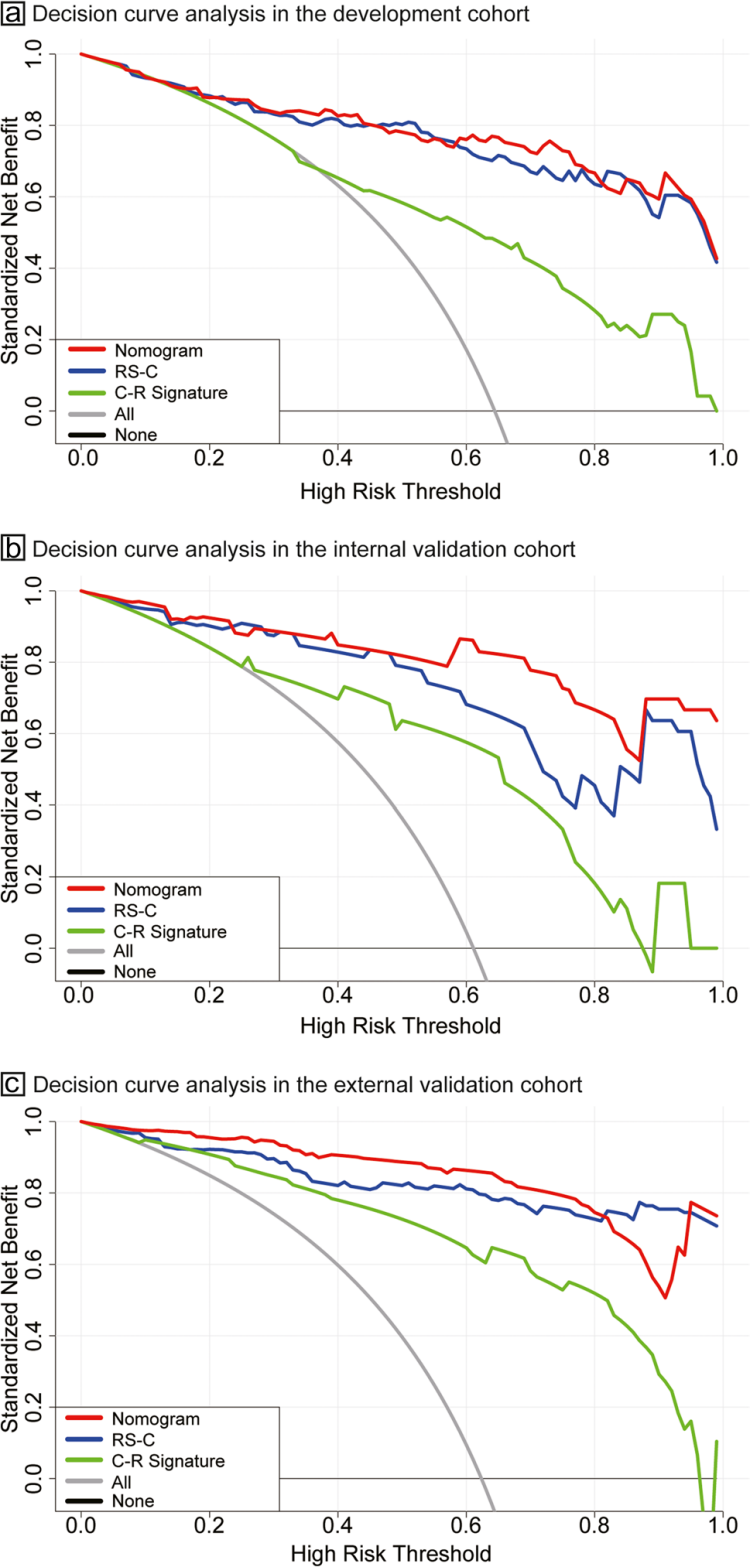
Decision curve analysis for the nomogram and signatures in the development and validation cohorts. Decision curve analysis for the nomogram and signatures in the development (**a**), internal (**b**), and external (**c**) validation cohorts. DCA, decision curve analysis; RS-C, combined radiomic signature selected from the nodular area and perinodular area; C-R, clinical–radiological

DCAs (Fig. 5) were used to assess the utility of the three predictive models by calculating the net benefit at various probability thresholds. According to the decision curves, the radiomic signature showed more benefit than the clinical–radiological signature in predicting the risk of the interstitial invasion when the probability threshold in the clinical decision of a patient or physician was above 0.2 in the development cohort. The nomogram line achieved the highest clinical net benefit across the entire range of threshold probabilities in three cohorts, which indicated that the nomogram was a reliable clinical tool to predict pathology invasiveness.

## Discussion

In this multicenter study, we built and validated a radiomic nomogram to distinguish PILs from early-stage pulmonary interstitium ILs preoperatively. The nomogram incorporated radiomic signatures selected from the nodular and perinodular areas and the clinical–radiological signature and performed well in the development and validation cohorts. The low AIC in the nomogram demonstrated the good quality of this available tool. The DCAs indicated that the nomogram is a reliable clinical treatment decision support tool to predict pulmonary interstitial invasion for patients with a solitary pulmonary nodule.

This research describes some important radiological characteristics that contribute to the differential diagnosis of SPN. The nodule with part-solid/solid density, pleural retraction, irregular shape, and blurred margins had a higher risk for malignancy, consistent with the radiologists’ experience. Previous researches [26, 27] have used nodule size and CTR to distinguish PILs from ILs. However, they were not included in the final predictive nomogram in our study.

We found considerable reliability of the radiomic features in the repeatability study. Overall, more than 83% (10/12) of the radiomic features achieve an excellent intrarater reliability (ICC ≥ 0.81) in the RS-C. To a certain extent, it can reflect the stability and generalization of the nomogram, which is mainly composed of the combined radiomic signature. Through commonly used and simple metrics, first-order statistics explain the distribution of voxel intensities inside the image region defined by the mask. GLCM is a statistical texture analysis method that evaluates the spatial relationship between pixels and determines how frequently a particular combination of pixels appears in an image. The radiomic features, including the sum average in the GLCM category and uniformity in the first-order category, were also reported to differentiate invasive pulmonary adenocarcinoma from PILs in a previous study [28]. The GLSZM provides information on the size of homogeneous zones for each gray level in three dimensions. Nearly half (5/12) of the combined radiomic signature was related to the GLCM and GLSZM categories and is stable by changes in the ROIs [29]. Although not statistically significant according to the DeLong test, RS2 can numerically distinguish PILs from early-stage pulmonary interstitium ILs and showed better performance than RS1 in all cohorts. Moreover, the lower AIC in RS2 indicates that its model quality is better than that of RS1. These findings may be due to more unstable features in the focal area than perinodular ones [29].

We constructed the first radiomic model combined with a 5-mm perinodular radiomic signature for the early diagnosis of pathological invasiveness. The predictive reproducibility of the models was evaluated in this multicentric study. Previous studies classified MIA as a benign or preinvasive pulmonary nodule because it has a good prognosis after surgical treatment as AIS [5]. Unlike other studies conducted by She et al [30] and Xu et al [31], we prefer to regard MIA as early-stage pulmonary malignant lesions because the difference between AIS and MIA lies in the microenvironment [30], especially in the expression level of laminin-5 [32–34] and the frequency of tumor protein p53 gene (TP53) mutations [35–37].

It is difficult for radiologists or thoracic surgeons to differentiate from PILs to ILs in pGGNs and PSNs. On the one hand, the surgical treatment strategies for patients with high-risk pulmonary nodules remain a massive challenge as the histopathologic definition is difficult to make before an operation. On the other hand, the entire histologic sampling of the tumor is required to diagnose the AIS or MIA, which may prolong the procedure time and lead to inappropriate surgical decision-making. In our study, the subgroup analysis shows good discrimination of the nomogram in the pGGNs and PSNs. A nomogram may determine whether surgical treatment or conservative surveillance is required and recommend management strategies for patients with nodules diagnosed as ILs.

The relevance of biological importance to the distribution of immune cells in the perinodular area has already been demonstrated [18]. The combination of intratumoral and peritumoral features proved useful in predicting the complete pathological response and lymph node metastasis, and identifying molecular subtypes [38–40]. Several studies demonstrated the added value of using radiomic features from perinodular parenchyma to differentiate nodules in terms of potential malignancy, and the definition of perinodular area differs between studies [41, 42]. Beig et al conducted a study [19] that used 30-mm perinodular radiomic features to distinguish IAC from benign granulomas, and the most predictive features were within a 5-mm perinodular area. The same distance of the perinodular area was also used in the study conducted by Wu et al [43]. As Wu et al indicated, adding perinodular features did not improve the radiomic model performance. However, the radiomic model achieved a better predictive value in our study after combining with the radiomic signature selected from the perinodular area.

In this study, pathological findings were used as the gold standard rather than the consensus malignancy rating of each nodule used in other studies [44]. This retrospective study was restricted to only suspected malignant nodules (PILs and ILs) and excluded some benign pulmonary lesions (tuberculosis or granulomas) to simulate the most likely clinical situation. Additionally, to improve the reliability of the results and enhance the homogeneity of the population, this multicenter study focused on patients with a solitary pulmonary nodule and excluded multiple pulmonary nodules.

Limitations of the study included its retrospective nature and the variation in the research period among the multiple centers, which prevented some clinical factors from being obtained, and a certain bias and heterogeneity may have existed in the study. Second, the CT acquisition protocol (i.e., image thickness) was not unified among all patients in the three centers. A standard process on radiomic features was performed to alleviate this problem, and the nomogram finally performed well in the external validation group. This finding indicated that the nomogram has good universality and is worthy of clinical application. Third, owing to the limitation of data, we did not have transcriptomics and mutation-sequencing data. Therefore, we could not further explain the relevant mechanism between radiomics and the tumor microenvironment. In the future, prospective, high-quality research with a larger population is still required to verify our results further.

## Conclusion

The perinodular radiomic signature improved the distinction between pulmonary interstitium ILs and PILs when combined with the nodular radiomic signature. This study demonstrated that a nomogram constructed by identifying the clinical–radiological signature and the combined radiomic signature has the potential to be an easy-to-use, non-invasive preoperative biomarker to precisely predict pathological invasiveness and add diagnostic value to clinical decisions for optimal intervention benefit.

## Supplementary Information

Below is the link to the electronic supplementary material.

Supplementary file1 (DOCX 3687 KB)

## Abbreviations

AIC: Akaike information criterion
AUC: Area under the receiver operating characteristics curve
CI: Confidence interval
C-R: Clinical–radiological
DCA: Decision curve analysis
GLCM: Gray-level co-occurrence matrix
GLDM: Gray-level dependence matrix
GLSZM: Gray-level size zone matrix
ILs: Invasion lesions
PILs: Pre-invasive lesions
RS1: Radiomic signature selected from the nodular area
RS2: Radiomic signature selected from the perinodular area
RS-C: Combined radiomic signature selected from the nodular area and perinodular area

## Declarations

### Guarantor:

The scientific guarantor of this publication is Prof. Zhou.

### Statistics and Biometry:

One of the authors (D. Chen) has significant statistical expertise.

### Methodology

• Retrospective • Diagnostic study • Multicenter study • Machine Learning Approach

## References

[1] The National Lung Screening Trial Research Team (2011). Reduced lung-cancer mortality with low-dose computed tomographic screening. N Engl J Med.

[2] MacMahon H, Naidich DP, Goo JM (2017). Guidelines for management of incidental pulmonary nodules detected on CT images: from the Fleischner Society 2017. Radiology.

[3] Callister MEJ, Baldwin DR, Akram AR (2015). British Thoracic Society guidelines for the investigation and management of pulmonary nodules: accredited by NICE. Thorax..

[4] Mets OM, de Jong PA, Chung K, Lammers J-WJ, van Ginneken B, Schaefer-Prokop CM (2016). Fleischner recommendations for the management of subsolid pulmonary nodules: high awareness but limited conformance – a survey study. Eur Radiol.

[5] Travis WD, Brambilla E, Noguchi M (2011). International Association for the Study of Lung Cancer/American Thoracic Society/European Respiratory Society: International Multidisciplinary Classification of Lung Adenocarcinoma: executive summary. Proc Am Thorac Soc.

[6] Borczuk AC, Qian F, Kazeros A (2009). Invasive size is an independent predictor of survival in pulmonary adenocarcinoma. Am J Surg Pathol.

[7] Zhang J, Wu J, Tan Q, Zhu L, Gao W (2013). Why do pathological stage IA lung adenocarcinomas vary from prognosis?: a clinicopathologic study of 176 patients with pathological stage IA lung adenocarcinoma based on the IASLC/ATS/ERS classification. J Thorac Oncol.

[8] Ost D, Fein A (2000). Evaluation and management of the solitary pulmonary nodule. Am J Respir Crit Care Med.

[9] Causey JL, Zhang J, Ma S (2018). Highly accurate model for prediction of lung nodule malignancy with CT scans. Sci Rep.

[10] Chae H-D, Park CM, Park SJ, Lee SM, Kim KG, Goo JM (2014). Computerized texture analysis of persistent part-solid ground-glass nodules: differentiation of preinvasive lesions from invasive pulmonary adenocarcinomas. Radiology.

[11] Bi WL, Hosny A, Schabath MB (2019). Artificial intelligence in cancer imaging: clinical challenges and applications. CA Cancer J Clin.

[12] Feng B, Chen X, Chen Y (2020). Solitary solid pulmonary nodules: a CT-based deep learning nomogram helps differentiate tuberculosis granulomas from lung adenocarcinomas. Eur Radiol.

[13] Chen S, Qin J, Ji X (2017). Automatic scoring of multiple semantic attributes with multi-task feature leverage: a study on pulmonary nodules in CT images. IEEE Trans Med Imaging.

[14] Lambin P, Rios-Velazquez E, Leijenaar R (2012). Radiomics: extracting more information from medical images using advanced feature analysis. Eur J Cancer.

[15] Gillies RJ, Kinahan PE, Hricak H (2016). Radiomics: images are more than pictures, they are data. Radiology.

[16] Yang L, Yang J, Zhou X (2019). Development of a radiomics nomogram based on the 2D and 3D CT features to predict the survival of non-small cell lung cancer patients. Eur Radiol.

[17] Song SH, Ahn JH, Lee HY (2016). Prognostic impact of nomogram based on whole tumour size, tumour disappearance ratio on CT and SUVmax on PET in lung adenocarcinoma. Eur Radiol.

[18] Beig N, Khorrami M, Alilou M (2019). Perinodular and intranodular radiomic features on lung CT images distinguish adenocarcinomas from granulomas. Radiology.

[19] Banat G-A, Tretyn A, Pullamsetti SS et al (2015) Immune and inflammatory cell composition of human lung cancer stroma. PLoS One 10(9):e0139073. 10.1371/journal.pone.0139073 10.1371/journal.pone.0139073 PMC4587668 26413839

[20] Nishino M (2019). Perinodular radiomic features to assess nodule microenvironment: does it help to distinguish malignant versus benign lung nodules?. Radiology.

[21] Christiansen A, Detmar M (2011). Lymphangiogenesis and cancer. Genes Cancer.

[22] van Griethuysen JJM, Fedorov A, Parmar C (2017). Computational radiomics system to decode the radiographic phenotype. Cancer Res.

[23] Goldstraw P, Chansky K, Crowley J (2016). The IASLC Lung Cancer Staging Project: proposals for revision of the TNM stage groupings in the forthcoming (eighth) edition of the TNM classification for lung cancer. J Thorac Oncol.

[24] Akaike H (1998) Information theory and an extension of the maximum likelihood principle. In: Parzen E, Tanabe K, Kitagawa G (eds) Selected papers of Hirotugu Akaike. Springer Series in Statistics (Perspectives in Statistics). Springer, New York, NY. 10.1007/978-1-4612-1694-0_15

[25] Vickers AJ, Elkin EB (2006). Decision curve analysis: a novel method for evaluating prediction models. Med Decis Making.

[26] Luo T, Xu K, Zhang Z (2019). Radiomic features from computed tomography to differentiate invasive pulmonary adenocarcinomas from non-invasive pulmonary adenocarcinomas appearing as part-solid ground-glass nodules. Chin J Cancer Res.

[27] Lee SM, Park CM, Goo JM, Lee H-J, Wi JY, Kang CH (2013). Invasive pulmonary adenocarcinomas versus preinvasive lesions appearing as ground-glass nodules: differentiation by using CT features. Radiology.

[28] Li W, Wang X, Zhang Y (2018). Radiomic analysis of pulmonary ground-glass opacity nodules for distinction of preinvasive lesions, invasive pulmonary adenocarcinoma and minimally invasive adenocarcinoma based on quantitative texture analysis of CT. Chin J Cancer Res.

[29] Tunali I, Hall LO, Napel S (2019). Stability and reproducibility of computed tomography radiomic features extracted from peritumoral regions of lung cancer lesions. Med Phys.

[30] She Y, Zhang L, Zhu H (2018). The predictive value of CT-based radiomics in differentiating indolent from invasive lung adenocarcinoma in patients with pulmonary nodules. Eur Radiol.

[31] Wu L, Gao C, Xiang P, Zheng S, Pang P, Xu M (2020). CT-imaging based analysis of invasive lung adenocarcinoma presenting as ground glass nodules using peri- and intra-nodular radiomic features. Front Oncol.

[32] Naito M, Aokage K, Saruwatari K (2016). Microenvironmental changes in the progression from adenocarcinoma in situ to minimally invasive adenocarcinoma and invasive lepidic predominant adenocarcinoma of the lung. Lung Cancer.

[33] Patarroyo M, Tryggvason K, Virtanen I (2002). Laminin isoforms in tumor invasion, angiogenesis and metastasis. Semin Cancer Biol.

[34] Moriya Y, Niki T, Yamada T, Matsuno Y, Kondo H, Hirohashi S (2001). Increased expression of laminin-5 and its prognostic significance in lung adenocarcinomas of small size: an immunohistochemical analysis of 102 cases. Cancer.

[35] Zhang C, Zhang J, Xu F-P (2019). Genomic landscape and immune microenvironment features of preinvasive and early invasive lung adenocarcinoma. J Thorac Oncol.

[36] Yim J, Zhu L-C, Chiriboga L, Watson HN, Goldberg JD, Moreira AL (2007). Histologic features are important prognostic indicators in early stages lung adenocarcinomas. Mod Pathol.

[37] Nakanishi H, Matsumoto S, Iwakawa R (2009). Whole genome comparison of allelic imbalance between noninvasive and invasive small-sized lung adenocarcinomas. Cancer Res.

[38] Braman NM, Etesami M, Prasanna P et al (2017) Intratumoral and peritumoral radiomics for the pretreatment prediction of pathological complete response to neoadjuvant chemotherapy based on breast DCE-MRI. Breast Cancer Res 19(1):1–14. 10.1186/s13058-017-0846-1 10.1186/s13058-017-0846-1 PMC5437672 28521821

[39] Braman N, Prasanna P, Whitney J (2019). Association of peritumoral radiomics with tumor biology and pathologic response to preoperative targeted therapy for HER2 (ERBB2) –positive breast cancer. JAMA Netw Open.

[40] Wang X, Zhao X, Li Q (2019). Can peritumoral radiomics increase the efficiency of the prediction for lymph node metastasis in clinical stage T1 lung adenocarcinoma on CT?. Eur Radiol.

[41] Levman JED, Martel AL (2011). A margin sharpness measurement for the diagnosis of breast cancer from magnetic resonance imaging examinations. Acad Radiol.

[42] Uthoff J, Stephens MJ, Newell JD et al (2019) Machine learning approach for distinguishing malignant and benign lung nodules utilizing standardized perinodular parenchymal features from CT. Med Phys 46(7):3207–3216. 10.1002/mp.13592 10.1002/mp.13592 PMC6945763 31087332

[43] Wu G, Woodruff HC, Shen J (2020). Diagnosis of invasive lung adenocarcinoma based on chest CT radiomic features of part-solid pulmonary nodules: a multicenter study. Radiology.

[44] Ferreira JR, Oliveira MC, de Azevedo-Marques PM (2018). Characterization of pulmonary nodules based on features of margin sharpness and texture. J Digit Imaging.

