# Supplementary material for: Development and validation of a preoperative CT-based radiomic nomogram to predict pathology invasiveness in patients with a solitary pulmonary nodule: a machine learning approach, multicenter, diagnostic study
Source: Eur Radiol. 2021 Oct 16;32(3):1983–96. doi: 10.1007/s00330-021-08268-z (PMC8831242; doi:10.1007/s00330-021-08268-z)
Supplement: Supplementary file 1 — Supplementary file1 (DOCX 3687 KB) [file 330_2021_8268_MOESM1_ESM.docx]

**Supplementary Material**

**eFigure 1.** Identification of essential radiomic features of the nodular and perinodular area with LASSO regression analysis

**eFigure 2.** Radiomic score for patients in the development and validation cohorts

**eFigure 3.** ROC curves of radiomic and clinical-radiological signatures in the development and validation cohorts

**eFigure 4.** ROC curves of the nomogram in density subgroup analysis in three cohorts

**eFigure 5.** Calibration curves of radiomic nomogram in the development and validation cohorts

**eTable 1.** Information on the three institutions in this study

**eTable 2.** The CT protocol of the three independent centers.

**eTable 3.** Essential radiomic features and formula composition.

**eTable 4.** Radiomic score in the development cohort and two validation cohorts.

**eTable 5.** Intrarater agreement analysis of radiomic feature extraction.

**eTable 6.** Univariable analysis of characteristics in three cohorts.

**eTable 7.** Comparison of clinical-radiological and combined radiomic signatures by multivariate logistic regression analysis.

**eTable 8.** Density subgroup analysis of the nomogram in three cohorts.

**eTable 9.** Performance and goodness-of-fit evaluation of models.

**eTable 10.** Abbreviation table.

**eFigure 1. Identification of essential radiomic features of the nodular and perinodular area with LASSO regression analysis**


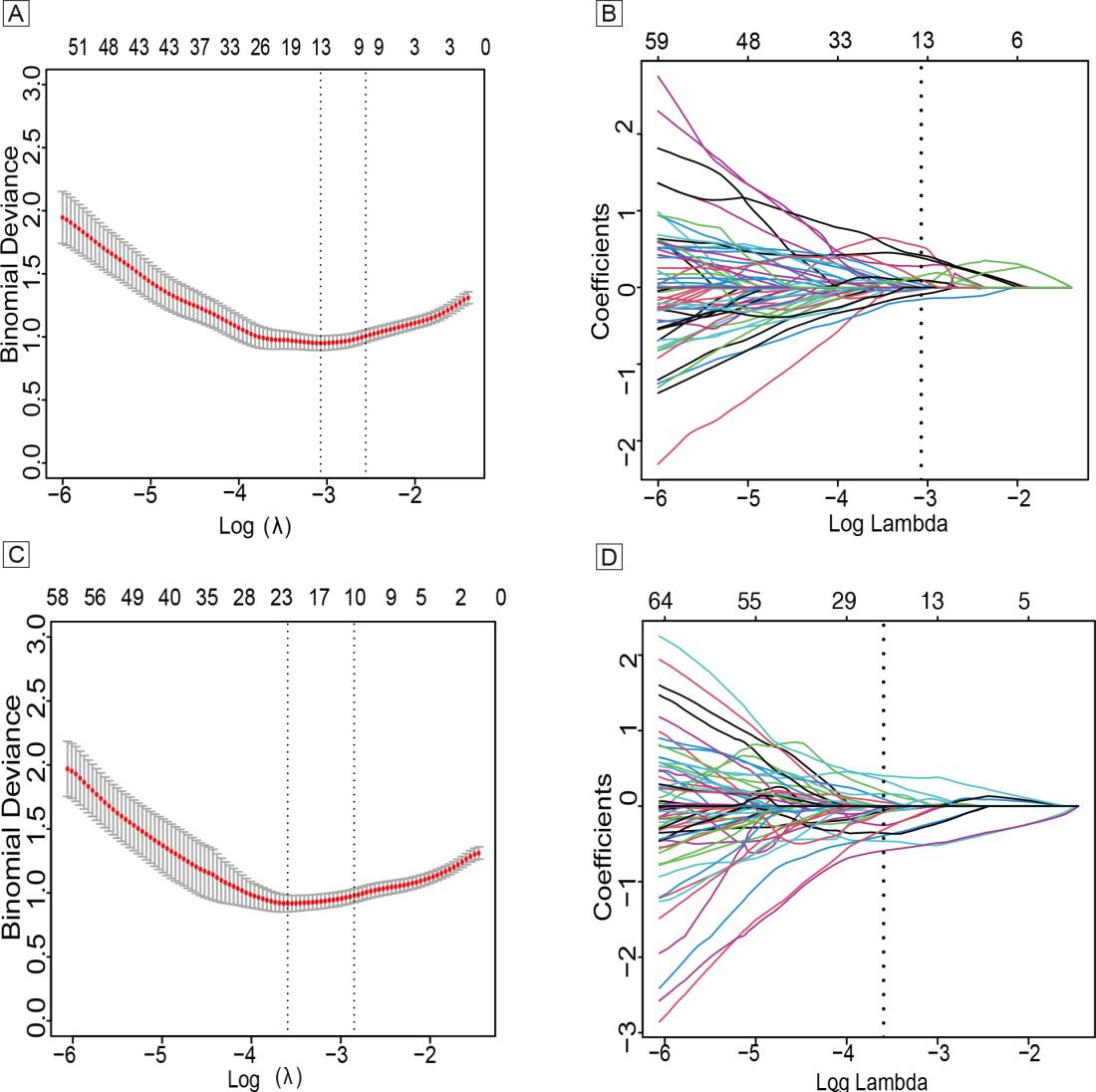


Select radiomic features from the nodular area (A–B) and perinodular area (C–D) with LASSO. LASSO, Least absolute shrinkage and selection operator.

**eFigure 2.** **Radiomic score for patients in the development and validation cohorts**


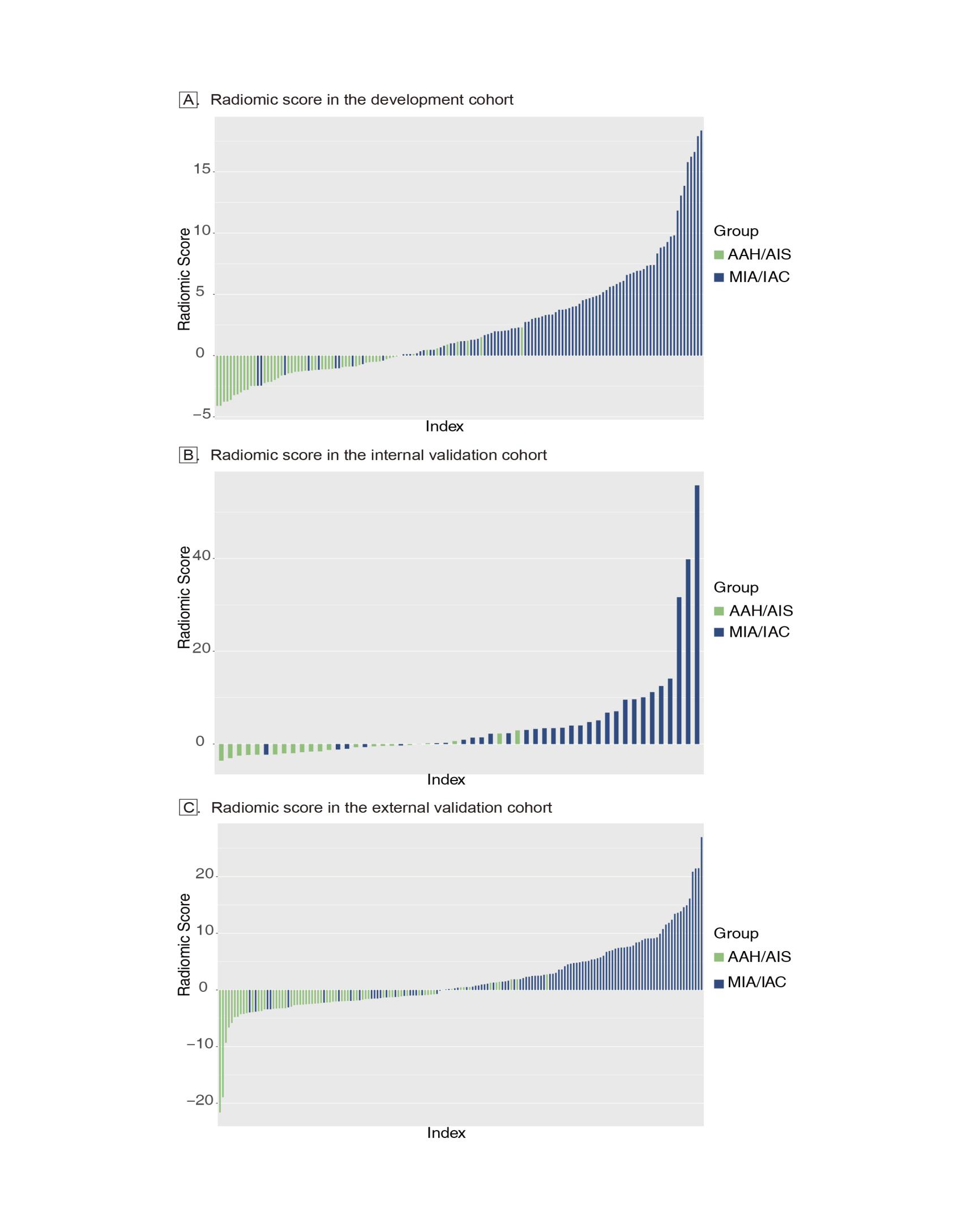


Radiomic score was calculated for each patient in the development cohort (A) and internal (B) and external (C) validation cohorts(*p*＜0.001 in the development cohort; *p*= 0.003 in the internal validation cohort; *p*＜0.001 in the external validation cohort. AAH, Atypical adenomatous hyperplasia; AIS, Adenocarcinoma in situ; MIA, Minimally invasive adenocarcinoma; IAC, Invasive adenocarcinoma.

**eFigure 3. ROC curves of radiomic and clinical-radiological signatures in the development and validation cohorts**
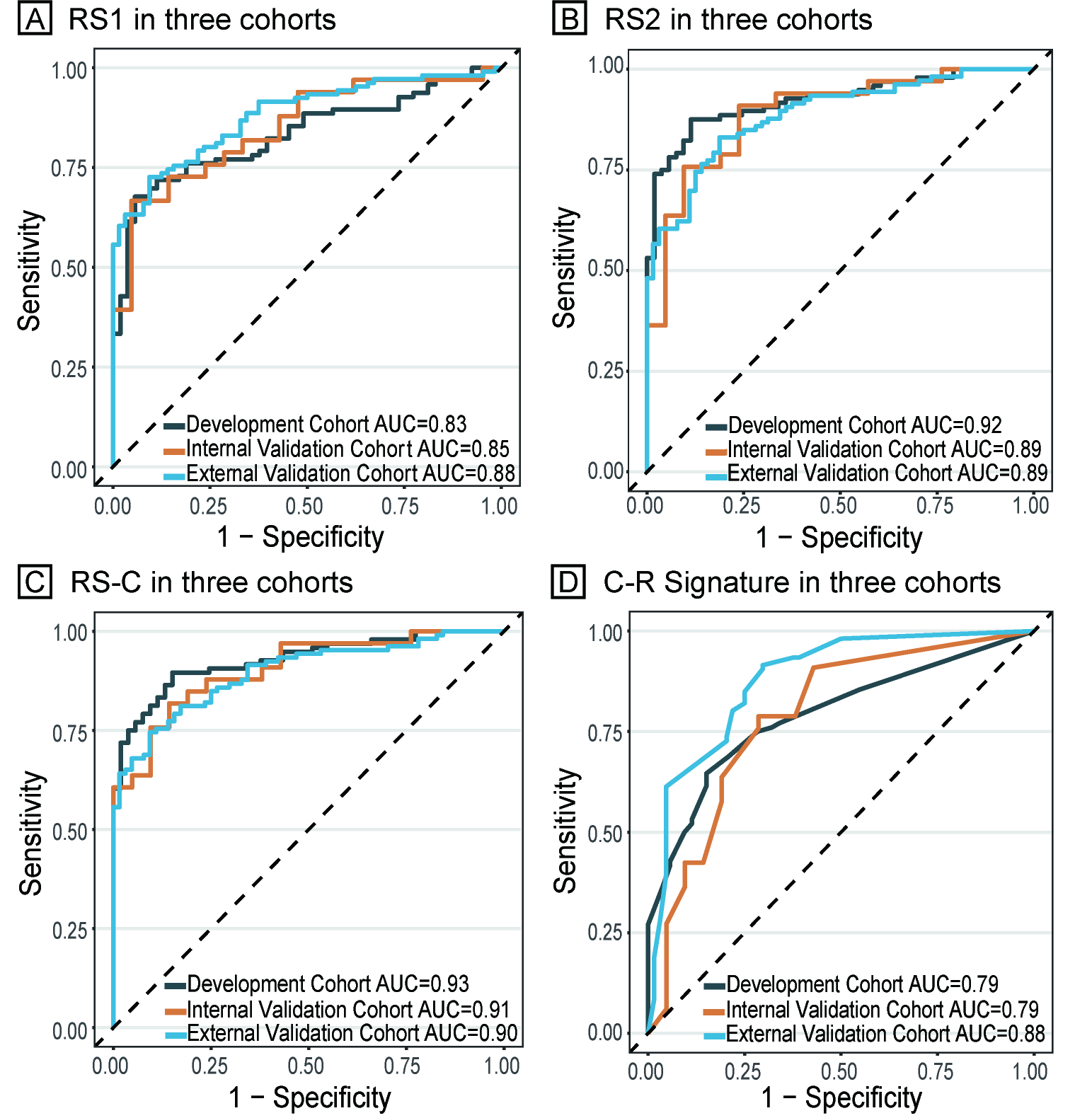


(A) ROC curves of RSI in the development and validation cohorts; (B) ROC curves of RS2 in the development and validation cohorts; (C) ROC curves of the combined radiomic signature in the development and validation cohorts; (D) ROC curves of the clinical-radiological signature in the development and validation cohorts; ROC, Receiver operating characteristic; RS1, Radiomic signature selected from the nodular area; RS2, Radiomic signature selected from the perinodular area; RS-C, Combined radiomic signature selected from the nodular area and perinodular area; C-R, Clinical-radiological.

**eFigure 4. ROC curves of the nomogram in density subgroup analysis in three cohorts**

**
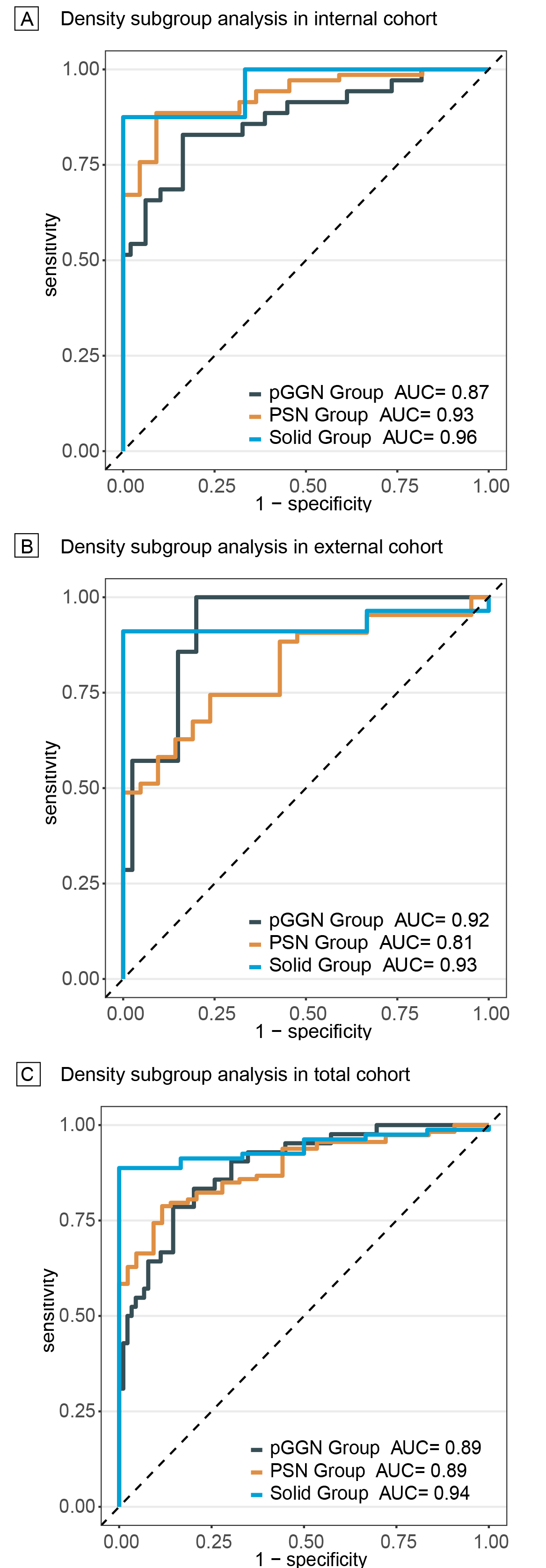
**

(A) ROC curves of the nomogram in density subgroup analysis in internal cohort; (B) ROC curves of the nomogram in density subgroup analysis in external cohort; (C) ROC curves of the nomogram in density subgroup analysis in total cohort; ROC, Receiver operating characteristic; pGGN, Pure ground-glass nodule; PSN, Part solid nodule.

**eFigure 5. Calibration curves of the radiomic nomogram in the development and validation cohorts**

**

**

Calibration curve of the radiomic nomogram showing the predicted versus actual probability for invasive pulmonary lesions in the development cohort and validation cohorts. Hosmer-Lemeshow test was applied in three cohorts (*p*= 0.97 in the development cohort; *p*= 0.97 in the internal validation cohort; *p*= 0.41 in the external validation cohort).

**eTable 1. Information on the three institutions in this study.**

| **Institution** | **Investigator in Charge** | **No. of Patients Enrolled** | **Treatment Period** |
| --- | --- | --- | --- |
| GDPH Center | Haiyu Zhou | 203 | March 1, 2015, to December 31, 2019 |
| SYSMH Center | Herui Yao | 63 | December 18,2012, to July 30,2019 |
| ZSLC Center | Herui Yao | 107 | January 1, 2019, to December 30, 2019 |
| GDPH Center, Guangdong Provincial people’ s Hospital; SYSMH Center, Sun Yat-Sen Memorial Hospital of Sun Yat-Sen University; ZSLC Center, Zhoushan Lung Cancer Institution; | | | |

**eTable 2. The CT protocol of the three independent centers.**

| **Parameters** | **GDPH Center** | **SYSMH Center** | **ZSLC Center** |
| --- | --- | --- | --- |
| CT version | GE Medical systems | GE Discovery 750HD | Aquilion 64 |
| CT tube voltage | 120 kVp | 120 kVp | 120kvp |
| CT tube current | 160 mAs | 160 mAs | 150mAs |
| CT rotation time | 0.8s | 0.6s | 0.8s |
| CT detector collimation | 64X0.625 | 64X0.625 | 64X0.625 |
| Contrast agent type | Iopamidol | Iopamidol | Iopamidol |
| Contrast agent concentration | 370 mg I/mL | 370 mg I/mL | 370mgI/mL |
| Contrast agent dosage | 90ml | 90ml | 90ml |
| Contrast agent infused rate | 3.0 mL/second | 4.0 mL/second | 3.5ml/second |
| Venous phase interval time | 60s | 40s | 40s |
| Image matrix | 512*512 | 512*512 | 512*512 |
| Field of view | 40 | 40 | 40 |
| Reconstruction image thickness | 1.250 mm | 1.250 mm | 1.0mm |
| GDPH Center, Guangdong Provincial people’ s Hospital; SYSMH Center, Sun Yat-Sen Memorial Hospital of Sun Yat-Sen University; ZSLC Center, Zhoushan Lung Cancer Institution; CT, Computed tomography. | | | |

**eTable 3. Essential radiomic features and formula composition.**

| **Model** | **Intercept/Feature Name** | **Regression coefficient** |
| --- | --- | --- |
| **RS1** | **Intercept=30.7450** | **β** |
| V16.x | original-Shape-LeastAxisLength | 0.5350 |
| V510.x | wavelet-HLH-GLCM-JointEnergy | -1.7751 |
| V530.x | wavelet-HLH-Firstorder-Uniformity | 2.0510 |
| V851.x | wavelet-LLL-GLSZM-LargeAreaHighGrayLevelEmphasis | 140.6303 |
| **RS2** | **Intercept=1.7687** | **β** |
| V23.y | original-Shape-Maximum2DDiameterColumn | 4.7728 |
| V24.y | original-Shape-Maximum2DDiameterRow | -1.3556 |
| V118.y | wavelet-HLL-GLDM-GrayLevelVariance | -26.3681 |
| V136.y | wavelet-HLL-GLCM-MaximumProbability | -0.6401 |
| V149.y | wavelet-HLL-GLCM-SumSquares | 27.4163 |
| V266.y | wavelet-LHL-Firstorder-Mean | 0.7258 |
| V691.y | wavelet-HHL-GLCM-SumAverage | -1.6534 |
| V808.y | wavelet-LLL-Firstorder-Skewness | -0.8694 |
| **RS-C** | **Intercept=4.2226** | **β** |
| V16.x | original-Shape-LeastAxisLength | -1.3592 |
| V510.x | wavelet-HLH-GLCM-JointEnergy | -0.9946 |
| V530.x | wavelet-HLH-Firstorder-Uniformity | 2.1861 |
| V851.x | wavelet-LLL-GLSZM-LargeAreaHighGrayLevelEmphasis | 9.9643 |
| V23.y | original-Shape-Maximum2DDiameterColumn | 5.9775 |
| V24.y | original-Shape-Maximum2DDiameterRow | -0.8391 |
| V118.y | wavelet-HLL- GLDM-GrayLevelVariance | -23.7268 |
| V136.y | wavelet-HLL-GLCM-MaximumProbability | -1.1408 |
| V149.y | wavelet-HLL-GLCM-SumSquares | 24.8522 |
| V266.y | wavelet-LHL-Firstorder-Mean | 0.7799 |
| V691.y | wavelet-HHL-GLCM-SumAverage | -1.0394 |
| V808.y | wavelet-LLL-Firstorder-Skewness | -0.8292 |
| **C-R Signature** | **Intercept=-0.6892** | **β** |
| Density | Part solid nodule | 0.3879 |
| Density | Solid nodule | 2.2454 |
| Pleura | Pleural retraction | 1.0099 |
| Shape | Irregular shape | 1.3027 |
| Margin | Blurred margin | 0.9744 |
| Abbreviations, β, Regression coefficient; RS1, Radiomic signature selected from the nodular area; RS2, Radiomic signature selected from the perinodular area; RS-C, Combined radiomic signature selected from the nodular area and perinodular area; C-R, Clinical-radiological. | | |

**eTable 4. Radiomic score in the development cohort and two validation cohorts.**

| **Group** | **Development Cohort** | | **Internal Validation Cohort** | | **External Validation Cohort** | |
| --- | --- | --- | --- | --- | --- | --- |
|  | **Rad-score** | ***p* value** | **Rad-score** | ***p* value** | **Rad-score** | ***p* value** |
| PILs | -1.24 | ＜0.001** | -1.06 | 0.003** | -2.59 | ＜0.001** |
| ILs | 7.28 |  | 7.45 |  | 8.34 |  |
| Abbreviations, PILs: Pre-invasive lesions; ILs: Invasive lesions; Rad-score, Radiomic score for combined radiomic signature; *, Significant at *p*＜0.05; **, Significant at *p*＜0.005. | | | | | | |

**eTable 5 Intrarater agreement analysis of radiomic feature extraction**

| **Feature** | **ICC** | **95%CI** | ***p* value** | **Agreement Level** |
| --- | --- | --- | --- | --- |
| V23.y | 0.96 | 0.908-0.979 | ＜0.001** | Excellent |
| V24.y | 0.93 | 0.863-0.969 | ＜0.001** | Excellent |
| V118.y | 0.82 | 0.643-0.910 | ＜0.001** | Excellent |
| V136.y | 0.98 | 0.949-0.989 | ＜0.001** | Excellent |
| V149.y | 0.77 | 0.558-0.885 | ＜0.001** | good |
| V266.y | 0.89 | 0.785-0.950 | ＜0.001** | Excellent |
| V691.y | 0.88 | 0.749-0.940 | ＜0.001** | Excellent |
| V808.y | 0.74 | 0.521-0.872 | ＜0.001** | good |
| V16.x | 0.99 | 0.981-0.996 | ＜0.001** | Excellent |
| V510.x | 0.94 | 0.877-0.972 | ＜0.001** | Excellent |
| V530.x | 0.92 | 0.841-0.963 | ＜0.001** | Excellent |
| V851.x | 0.99 | 0.999-0.999 | ＜0.001** | Excellent |
| Abbreviations, ICC, Intraclass correlation efficient; CI, Confidence interval; **, Significant at *p*＜0.005. | | | | |

**eTable 6. Univariable analysis of characteristics in three cohorts.**

| **Variable** | **Development Cohort**  **(N=149)** | | ***p* value** | **Internal Validation Cohort (N=54)** | | ***p* value** | **Internal Total**  **Cohort (N=203)** | | ***p* value** |
| --- | --- | --- | --- | --- | --- | --- | --- | --- | --- |
|  | **PILs**  **(N=53)** | **ILs**  **(N=96)** |  | **PILs**  **(N=21)** | **ILs**  **(N=33)** |  | **PILs**  **(N=74)** | **ILs**  **(N=129)** |  |
| Age at diagnosis, years,  No. (%) |  |  | 0.57 |  |  | 1.00 |  |  | 0.59 |
| Mean (SD) | 59.0 (12.9) | 58.5 (12.6) |  | 54.0 (11.8) | 55.0(12.2) |  | 56.1 (12.6) | 57.1(12.5) |  |
| Median (IQR) | 56.7  [48.0, 64.0] | 58.0  [50.8, 66.0] |  | 54.7  [47.0, 61.0] | 54.7  [44.0, 65.0] |  | 58.0  [47.0, 63.7] | 57.0  [49.0, 66.0] |  |
| Range | [31.0, 86.0] | [29.0, 84.0] |  | [27.0, 76.0] | [33.0, 80.0] |  | [27.0, 86.0] | [29.0, 84.0] |  |
| ＜60y | 33 (60.0) | 55 (59.8) |  | 9 (47.4) | 16 (43.2) |  | 42 (56.8) | 71 (55.0) |  |
| ≥60y | 22 (40.0) | 37 (40.2) |  | 10 (52.6) | 21 (56.8) |  | 32 (43.2) | 58 (45.0) |  |
| Gender, No. (%) |  |  |  |  |  |  |  |  |  |
| Male | 18 (34.0) | 39 (40.6) | 0.48 | 11 (52.4) | 12 (36.4) | 0.27 | 29 (39.2) | 51 (39.5) | 1.0 |
| Female | 35 (66.0) | 57 (59.4) |  | 10 (47.6) | 21 (63.6) |  | 45 (60.8) | 78 (60.5) |  |
| BMI, No. (%) |  |  |  |  |  |  |  |  |  |
| ＜18.5 | 5 (9.4) | 4 (4.2) | 0.33 | 0 (0) | 1 (3.0) | 0.16 | 5(6.8) | 5(3.9) | 0.44 |
| 18.5-23.9 | 27 (50.9) | 60 (62.5) |  | 16 (76.2) | 18 (54.5) |  | 43(58.1) | 78(60.5) |  |
| ＞24 | 8 (15.1) | 16 (16.7) |  | 2 (9.5) | 9 (27.3) |  | 10(13.5) | 25(19.4) |  |
| Smoking status, No. (%) |  |  |  |  |  |  |  |  |  |
| Non-  smokers | 40 (75.5) | 83 (86.5) | 0.12 | 16 (76.2) | 29 (87.9) | 0.39 | 56(75.7) | 112(86.8) | 0.10 |
| Former smoker | 3 (5.7) | 6 (6.2) |  | 3 (14.3) | 1 (3.0) |  | 6(8.1) | 7(5.4) |  |
| Current Smokers | 10 (18.9) | 7 (7.3) |  | 2 (9.5) | 3 (9.1) |  | 12(16.2) | 10(7.8) |  |
| Primary site of tumor, No. (%) |  |  |  |  |  |  |  |  |  |
| LLL | 8 (15.1) | 15 (15.6) | 0.80 | 3 (14.3) | 6 (18.2) | 0.84 | 11 (14.9) | 21 (16.3) | 0.97 |
| LUL | 10 (18.9) | 25 (26.0) |  | 7 (33.3) | 6 (18.2) |  | 17 (23.0) | 31 (24.0) |  |
| RLL | 11 (20.8) | 16 (16.7) |  | 4 (19.0) | 8 (24.2) |  | 15 (20.3) | 24 (18.6) |  |
| RML | 3 (5.7) | 8 (8.3) |  | 1 (4.8) | 2 (6.1) |  | 4 (5.4) | 10 (7.8) |  |
| RUL | 21 (39.6) | 32 (33.3) |  | 6 (28.6) | 11 (33.3) |  | 27 (36.5) | 43 (33.3) |  |
| Pathology diagnosis, No. (%) |  |  |  |  |  |  |  |  |  |
| AAH | 11(20.8) | 0(0) |  | 2(10) | 0(0) |  | 13(17.6) | 0(0) |  |
| AIS | 42(79.2) | 0(0) |  | 19(90) | 0(0) |  | 61(82.4) | 0(0) |  |
| MIA | 0(0) | 28(29.2) |  | 0(0) | 4(12.1) |  | 0(0) | 32(24.8) |  |
| IAC | 0(0) | 68(70.8) |  | 0(0) | 29(87.9) |  | 0(0) | 97(75.2) |  |
| Density, No. (%) |  |  |  |  |  |  |  |  |  |
| pGGN | 31 (58.5) | 27 (28.1) | < 0.001** | 17 (81.0) | 9 (27.3) | <.001** | 48 (64.9) | 36 (27.9) | < .001** |
| PSN | 19 (35.8) | 50 (52.1) |  | 3 (14.3) | 20 (60.6) |  | 22 (29.7) | 70 (54.3) |  |
| Solid | 3 (5.7) | 19 (19.8) |  | 1 (4.8) | 4 (12.1) |  | 4 (5.4） | 23 (17.8) |  |
| CTR of the PSNs，No. (%) | 18 | 48 |  | 3 | 20 |  | 21 | 68 |  |
| ＜0.5 | 16(84.2) | 12(24.0） | < 0.001** | 3(100.0) | 10(50.0) | 0.1 | 19(86.4) | 22(31.4) | < .001** |
| ≥0.5 | 3(15.8) | 38(76.0) |  | 0(0.0) | 10(50.0) |  | 3(9.5) | 48(68.6) |  |
| Size, mm  No. (%) |  |  |  |  |  |  |  |  |  |
| Mean (SD) | 10.5(7.9) | 11.9(7.9) |  | 9.8(8.4) | 12.0(9.3) |  | 10.3(8.0) | 11.9(8.2) |  |
| Median (IQR) | 8.0  [5.0, 17.0] | 10.0  [6.0, 17.0] |  | 8.0  [2.0, 18.0] | 10.0  [2.0,20.0] |  | 8.0  [3.0, 17.5] | 10.0  [6.0, 18.0] |  |
| Range | [1.0,30.0] | [1.0,30.0] |  | [1.0,30.0] | [1.0,30.0] |  | [1.0, 30.0] | [1.0, 30.0] |  |
| 0-10 | 33 (62.3) | 53 (55.2) | 0.43 | 15 (71.4) | 17 (51.5) | 0.45 | 48(64.9) | 70(54.3) | 0.21 |
| 10-20 | 15 (28.3) | 26 (27.1) |  | 4 (19.0) | 10 (30.3) |  | 19(25.7) | 36(27.9) |  |
| 20-30 | 5 (9.4) | 17 (17.7) |  | 2 (9.5) | 6 (18.2) |  | 7(9.5) | 23(17.8) |  |
| Pleural retraction, No. (%) |  |  |  |  |  |  |  |  |  |
| No | 48 (90.6) | 62 (64.6) | < 0.001** | 18 (85.7) | 20 (60.6) | 0.07 | 66 (89.2) | 82 (63.6) | < 0.001** |
| Yes | 5 (9.4) | 34 (35.4) |  | 3 (14.3) | 13 (39.4) |  | 8 (10.8) | 47 (36.4) |  |
| Shape,  No. (%) |  |  |  |  |  |  |  |  |  |
| Round or oval | 43 (81.1) | 41 (42.7) | < 0.001** | 15 (71.4) | 11 (33.3) | 0.01* | 58 (78.4) | 52 (40.3) | < 0.001** |
| Irregular | 10 (18.9) | 55 (57.3) |  | 6 (28.6) | 22 (66.7) |  | 16 (21.6) | 77 (59.7) |  |
| Bubble sign,  No. (%) |  |  |  |  |  |  |  |  |  |
| No | 50 (94.3) | 80 (83.3) | 0.07 | 19 (90.5) | 27 (81.8) | 0.46 | 69 (93.2) | 107 (82.9) | 0.05 |
| Yes | 3 (5.7) | 16 (16.7) |  | 2 (9.5) | 6 (18.2) |  | 5 (6.8) | 22 (17.1) |  |
| Lobulated border,  No. (%) |  |  |  |  |  |  |  |  |  |
| No | 45 (84.9) | 52 (54.2) | < 0.001** | 15 (71.4) | 15 (45.5) | 0.09 | 60 (81.1) | 67 (51.9) | < 0.001** |
| Yes | 8 (15.1) | 44 (45.8) |  | 6 (28.6) | 18 (54.5) |  | 14 (18.9) | 62 (48.1) |  |
| Clear margin, No. (%) |  |  |  |  |  |  |  |  |  |
| No | 48 (90.6) | 70 (72.9) | 0.01* | 19 (90.5) | 25 (75.8) | 0.28 | 67 (90.5) | 95 (73.6) | 0.004** |
| Yes | 5 (9.4) | 26 (27.1) |  | 2 (9.5) | 8 (24.2) |  | 7 (9.5) | 34 (26.4) |  |
| Abbreviations, SD, Standard deviation; IQR, Interquartile range; PILs: Pre-invasive lesions; ILs: Invasive lesions; LLL, Left lower lobe; LUL, Left upper lobe; RLL, Right lower lobe; RML, Right middle lobe; RUL, Right upper lobe; CTR, Consolidation tumor ratio; pGGN, Pure ground-glass nodule; PSN, Part solid nodule; *, Significant at *p*＜0.05; **, Significant at *p*＜0.005; PILs, Pre-invasive lesions; ILs, Invasion lesions. | | | | | | | | | |

**eTable 7. Comparison of clinical-radiological and combined radiomic signatures by multivariate logistic regression analysis.**

| **Signature** | **Multivariate logistic regression analysis** | | |
| --- | --- | --- | --- |
|  | **β** | **OR (95 %CI)** | ***P value*** |
| Clinical-radiological Signature | 0.47 | 1.60 (1.03-2.59) | 0.04* |
| Combined Radiomic Signature | 0.89 | 2.43 (1.76-3.66) | < 0.001** |
| Abbreviations, OR, Odds ratio; CI, Confidence interval; β, Regression coefficient; *, Significant at *p*＜0.05; **, Significant at *p*＜0.005. | | | |

**eTable 8. Density subgroup analysis of the nomogram in three cohorts.**

| **Cohort** | **Subgroup** | **Signature Performance** | | | | | |
| --- | --- | --- | --- | --- | --- | --- | --- |
|  |  | **Sensitivity** | **Specificity** | **Accuracy** | **PPV** | **NPV** | **AUC (95%CI)** |
| Internal Cohort | pGGN | 0.83 | 0.84 | 0.87 | 0.78 | 0.87 | 0.87 (0.79-0.95) |
|  | PSN | 0.89 | 0.91 | 0.93 | 0.97 | 0.71 | 0.93 (0.88-0.98) |
|  | Solid | 0.88 | 0.99 | 0.89 | 0.99 | 0.5 | 0.96 (0.87-0.99) |
| External  Cohort | pGGN | 0.99 | 0.8 | 0.83 | 0.47 | 0.99 | 0.92 (0.84-0.99) |
|  | PSN | 0.74 | 0.76 | 0.75 | 0.87 | 0.59 | 0.81 (0.71-0.92) |
|  | Solid | 0.91 | 0.99 | 0.92 | 0.99 | 0.38 | 0.93 (0.86-0.99) |
| Total Cohort | pGGN | 0.79 | 0.85 | 0.83 | 0.72 | 0.89 | 0.89(0.83-0.95) |
|  | PSN | 0.79 | 0.88 | 0.81 | 0.95 | 0.61 | 0.89(0.83-0.94) |
|  | Solid | 0.89 | 0.99 | 0.90 | 0.99 | 0.4 | 0.94(0.89-0.99) |
| Abbreviations, PPV, Positive predictive values; NPV, Negative predictive values; AUC, Area under the receiver operating characteristics curve; CI, Confidence interval; pGGN, Pure ground-glass nodule; PSN, Part solid nodule. | | | | | | | |

**eTable 9. Performance and goodness-of-fit evaluation of models.**

| **Signature** | **AIC** | **DeLong Test (compared with Nomogram)** |
| --- | --- | --- |
| RS1 | 149.83 | 0.005* |
| RS2 | 117.62 | 0.18 |
| RS-C | 121.0 | 0.15 |
| C-R Signature | 159.36 | ＜0.001** |
| Nomogram | 121.68 | / |
| Abbreviations, AIC, Akaike information criterion; RS1, Radiomic signature selected from nodular area; RS2, Radiomic signature selected from perinodular area; RS-C, Combined radiomic signature selected from the nodular area and perinodular area; C-R, Clinical-radiological *, Significant at *p*＜0.05; **, Significant at *p*＜0.005. | | |

**eTable 10. Abbreviation table.**

| **Abbreviations** | **Meaning** |
| --- | --- |
| AIC | Akaike information criterion |
| AIS | Adenocarcinoma in situ |
| AUC | Area under the receiver operating characteristics curve |
| BTS | British Thoracic Society |
| CI | Confidence interval |
| CT | Computed tomography |
| C-R | Clinical-radiological |
| CTR | Consolidation tumor ratio |
| DCA | Decision curve analysis |
| GDPH | Guangdong Provincial People’s Hospital |
| GLCM | Gray Level Co-occurrence Matrix |
| GLDM | Gray Level Dependence Matrix |
| GLSZM | Gray Level Size Zone Matrix |
| IAC | Invasive adenocarcinoma |
| IASLC/ATS/ERS | International Association for the Study of Lung Cancer/American Thoracic Society/European Respiratory Society |
| ICC | Intraclass correlation efficient |
| IHC | Immunohistochemical |
| ILs | Invasion lesions |
| IQR | Interquartile range |
| LASSO | Least absolute shrinkage and selection operator |
| LDCT | Low-dose computed tomography |
| LLL | Left lower lobe |
| LUL | Left upper lobe |
| Lung-RADS | Lung CT Screening Reporting and Data System |
| LUSC | Lung squamous cell cancer |
| MIA | Minimally invasive adenocarcinoma |
| NGTDM | Neighbouring Gray Tone Difference Matrix |
| NPV | Negative predictive values |
| OR | Odds ratio |
| PACS | Picture Archiving and Communication System |
| pGGN | Pure ground-glass nodule |
| PILs | Pre-invasive lesions |
| PPV | Positive predictive values |
| PSN | Part solid nodule |
| Rad-score | Radiomic score |
| RLL | Right lower lobe |
| RML | Right middle lobe |
| ROC | Receiver operating characteristic |
| ROI | Regions of interest |
| RS-C | Combined radiomic signature selected from the nodular area and perinodular area |
| RS1 | Radiomic signature selected from the nodular area |
| RS2 | Radiomic signature selected from the perinodular area |
| RUL | Right upper lobe |
| SCLC | Small cell lung cancer |
| SD | Standard deviation |
| SPN | Solid pulmonary nodule |
| SYSMH | Sun Yat-Sen Memorial Hospital of Sun Yat-Sen University |
| TP53 | Tumor protein p53 gene |
| VOI | Volumes of interest |
| ZSLC | Zhoushan Lung Cancer Institution |
| β | Regression coefficient |
| * | Significant difference between two groups in cohorts(P＜.05) |
| ** | Significant difference between two groups in cohorts(*P*＜.005) |
